# A general model of intra-annual tree growth using dendrometer bands

**DOI:** 10.1002/ece3.1117

**Published:** 2014-12-21

**Authors:** Sean M McMahon, Geoffrey G Parker

**Affiliations:** Smithsonian Environmental Research CenterEdgewater, Maryland, USA

**Keywords:** Dendrometer bands, forest ecology, intra-annual growth, logistic function, maximum likelihood, optimization

## Abstract

Tree growth is an important indicator of forest health, productivity, and demography. Knowing precisely how trees' grow within a year, instead of across years, can lead to a finer understanding of the mechanisms that drive these larger patterns. The growing use of dendrometer bands in research forests has only rarely been used to measure growth at resolutions finer than yearly, but intra-annual growth patterns can be observed from dendrometer bands using precision digital calipers and weekly measurements. Here we present a workflow to help forest ecologists fit growth models to intra-annual measurements using standard optimization functions provided by the R platform. We explain our protocol, test uncertainty in parameter estimates with respect to sample sizes, extend the optimization protocol to estimate robust lower and upper annual diameter bounds, and discuss potential challenges to optimal fits. We offer R code to implement this workflow. We found that starting values and initial optimization routines are critical to fitting the best functional forms. After using a bounded, broad search method, a more focused search algorithm obtained consistent results. To estimate starting and ending annual diameters, we combined the growth function with early and late estimates of beginning and ending growth. Once we fit the functions, we present extension algorithms that estimate periodic reductions in growth, total growth, and present a method of controlling for the shifting allocation to girth during the growth season. We demonstrate that with these extensions, an analysis of growth response to weather (e.g., the water available to a tree) can be derived in a way that is comparable across trees, years, and sites. Thus, this approach, when applied across broader data sets, offers a pathway to build inference about the effects of seasonal weather on growth, size- and light-dependent patterns of growth, species-specific patterns, and phenology.

## Introduction

Growth can integrate important influences on an individual's fitness. Tree diameter growth correlates with biomass, and therefore carbon uptake, as well as with pathogen damage, nutrient availability, and the influence of climate on photosynthesis among others Barford et al. (2001). Growth is most often measured in annual censuses or longer, periods greater than the physiological processes operating. Developing a better sense of the mechanisms that determine growth requires more frequent measurements that can be used to develop models of the processes. For ecological inference, these models need to be synthesized across many individuals, populations, species, and sites, and so require a consistent method of extracting growth parameters.

Modeling how the subannual growth of individual trees has largely been the purview of physiological ecologists. Ecosystem ecologists have focused on measuring carbon movement into and out of forest systems using coarse spatial-scale instrumentation, such as eddy-flux towers. Demographic data on tree growth and mortality from plot networks have also been used to infer carbon stocks (e.g., (Chave et al. 2005; McMahon et al. 2010)). Carbon fluxes have been estimated from growth for plots with multiple censuses of bole diameter at breast height (DBH, or 1.34 m), but these have often been drawn from data with coarse temporal grain (i.e., census intervals often equal to or exceeding 5 years Condit (1998), so we miss the timescale of driving processes. This approach offers insight into the patterns of carbon stocks and flux, but offers limited inference on mechanisms, especially related to plant physiology.

Dendrometer bands, steel, or plastic bands that are permanently attached to trees with a spring can be measured easily with precision digital calipers, and offer a low-cost, accurate method of measuring tree diameter. They have most often been used for developing more precise estimates of annual growth, the core vital rate measurement for forest demography. Some examples of intra- annual uses of dendrometer bands include investigation of seasonal dynamics and growth phenology. Girardin et al. (2010) measured dendrometer bands every 3 months to capture seasonal changes in NPP, as did Palmer and Ogden (1983) who used monthly diameter measurements in kauri trees in New Zealand. More intensive measurement protocols have been used. Edwards and Hanson (1996) measured diameter biweekly and monthly, and Deslauriers et al. (2007) automated dendrometer bands to investigate 3 years of growth at 15-min intervals for four trees in the Italian Alps. These studies produced a more detailed pattern of tree growth, and Deslauriers et al. (2007) were able to estimate day of starting growth for their four trees. These studies demonstrate the importance of fine-scale analyses of growth, but none produce a way of assessing differences in growth that can be reproduced across many trees and species and sites. Here we propose an extension of these approaches, where a weekly or biweekly measuring protocol (as with Deslauriers et al. (2007)) is applied to many trees, with a fitting function that can be used to estimate annual growth, the shape of the growth function, and provide deviations from that function that indicate pauses in growth due to weather.

In 2010, we collected weekly measurements of dendrometer bands on ∼100 trees in a 16 ha Smithsonian forest dynamics plot at the Smithsonian Environmental Research Center, Edgewater, MD. This protocol produced a refined assessment of intra-annual tree growth, one that has showed, even by visual inspection, a remarkably precise description of tree diameter change the growing season, one that can show subtle, weekly, responses of tree growth to precipitation and temperatures. Our approach has been to use cases of weather-driven growth response to build a library of annual scenarios and fit functional forms to these data to be able to compare tree growth patterns within and among years.

**Figure 1:**
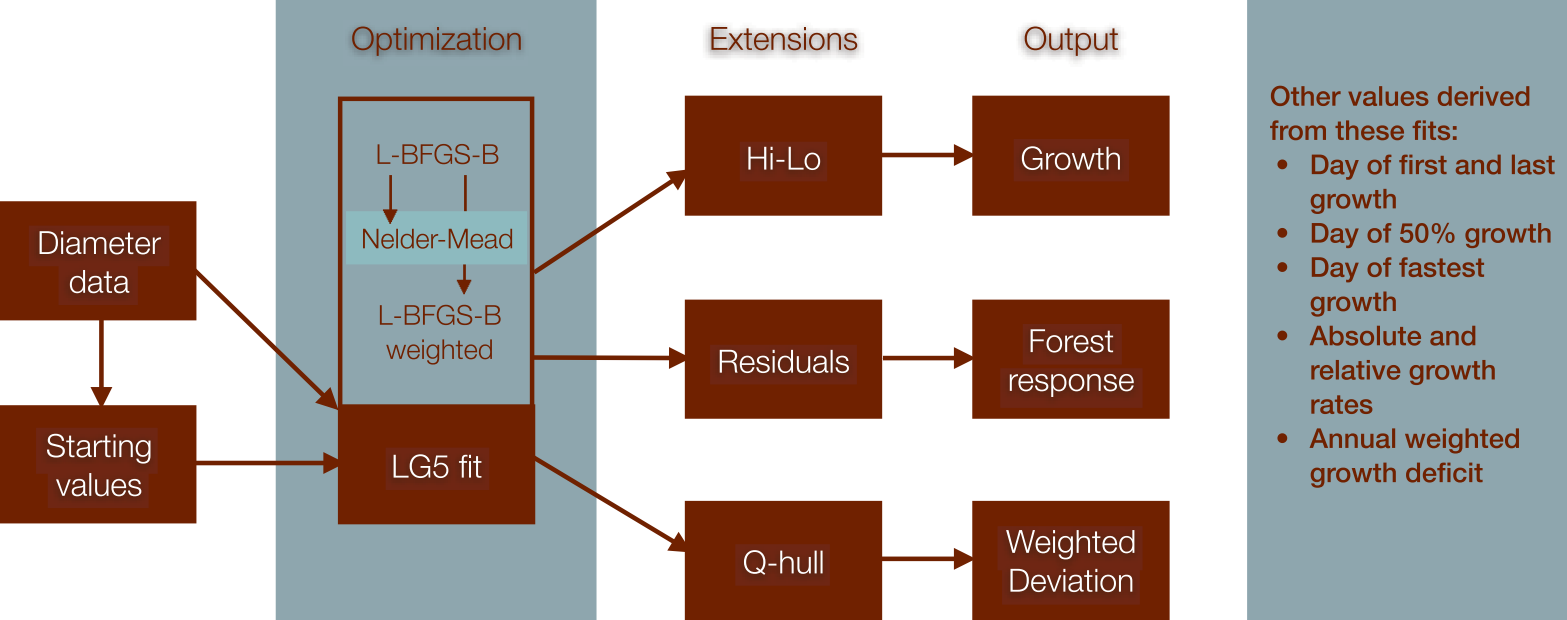
A diagram of the workflow for intra-annual diameter data.

The core of our workflow involves fitting a functional form to the time series of diameter measurements that captures the intra-annual process of individual tree growth (Fig. 1). Such a growth model reflects the allocation of carbon to bole biomass, which is fundamental to establishing a biological baseline process of plant growth (Paine et al. 2012). As such, deviations from the model may offer insight into when allocation is constrained by resource acquisition, such as during periods of high temperatures or drought. Inference about such fine-scale physiological dynamics is necessarily sensitive to the precision of the growth process model that determines, from measurement data, what the “expected” growth trajectory over a season. Important inference on biome response to climate requires developing a growth process model that works across studies for many trees at many sites over multiple years and thus modeling process requires more than fitting post hoc functions to individual data sets, but a complete approach that can be reproduced in diverse study systems.

The biggest challenge to fitting growth process models to single-tree data is the complex parameter space that can describe the growth function, especially when very small deviations from this function are being interpreted as environmental (biotic or abiotic) influences on an “expected” growth process. There are some key criteria we apply when searching for a growth function.

1. The function must be parametric. Nonparametric functions can produce very precise fits, but they cannot parsimoniously reflect the process of growth, that is, the fundamental goal of this workflow.
2. The fit needs to be repeatable. Through reliance on stochastic fitting algorithms (i.e., simulated annealing or Metropolis-Hastings), stochastic starting points (from random draws), or highly customized starting values (where tracking and comparing starting values from one tree to another can lead to ambiguous comparisons), we aimed to create a workflow that could be used on every tree in every year at every site and find the same optimal solution with similar precision.
3. The function can lead to ecologically and physiologically meaningful metrics. A goal of the overall workflow was to develop consistency in fitting functions and therefore maximize our ability to compare observations.

There are additional potential criteria for fitting, such as fit statistics and parsimony. We will address some of these in the discussion, but the items above we see as fundamental to the analysis of multiple growth trajectories, while other criteria are more subject to the goals and interests of particular studies. Overall, however, we selected a highly flexible function, the 5-parameter logistic function ([LG5], also known as the Richard's function (Oswald et al. 2012)), and a suite of potential optimization algorithms that are part of the optim() function in R base R Core Team (2014). We demonstrate that the LG5 function, and our final protocol, serves all three goals and offers robust results for a variety of data forms.

Because we understand the physiological processes that constrain growth, without even conducting an analysis, we can draw line by hand through data that gives a reasonable estimate of growth over time. Optimizing functions, conversely, are not informed by biology. For example, where we see a spring- time beginning of growth, an optimizing function sees a few observations. Where we see a progressive slowing of growth in autumn, an optimization procedure sees noise.

These challenges encouraged a workflow that appreciates the strengths and weaknesses of search algorithms while testing them for best fits. Specifically, we will here address (1) starting values, (2) identifying the optimizing function(s) that lead to best fits, and (3) analyses of the resulting optimization, including uncertainty, residuals, and output statistics. We also demonstrate a method to estimate the starting and ending diameter of the growing season – one that may not be captured by the estimated asymptote parameters in the logistic function.

## Methods

### Site and measurement protocol

In the fall of 2009, we installed dendrometer bands on 100 trees in the Smithsonian Institutions Global Earth Observatory (SIGEO) plot at the Smithsonian Environmental Research Center (SERC) (Latitude:38.889, Longitude:−76.559). The forest is a mature stand of trees of the tulip poplar (*Liriodendron tulipifera*) association (Brush et al. 1980). The data we use here contain tulip poplar, beech (*Fagus grandifolia*), sweetgum (*Liquidambar styraciflua*, and white oak (*Quercus alba*). The bands were measured approximately once a week through the growing season in 2010. Each band was measured in the morning using digital calipers. We used twenty trees from the 2010 growing season containing different species and size classes to develop the model workflow.

### The structure of the data

Dendrometer band data consist of a series of measurements of the band circumference over the course of the growing season. Using precision digital calipers, resolutions are typically ∼ 0.01 mm, while observational error (two standard deviations in measurements of the same tree) is around 0.03 mm (note that analog calipers have a precision of 0.1mm and will fail to provide data on growth response to climate that digital calipers will).

Two transformations of these gap data are helpful for analysis. First, changing the date of measurement into a day of the year allows for function fitting of the date as a continuous variable. This can be done using the julian() function in the chron package [not executed here]). For example, for a list of days in months in a particular year, the day of the year can be calculated as:

>doy <- julian (day, month, year,origin = c (1, 1,year))

Additionally, it can be worthwhile to translate the gap increment measured with calipers into units of dbh so that all further analyses are carried out on the appropriately scaled data. This merely takes the circumference of the band (which should be recorded when the band is installed) and relates gap size to circumference, with a division by *pi* (multiplied by 10 to convert the mm caliper measurement to cm DBH) producing diameter values.

A data sample from a single 19 cm dbh beech (*Fagus grandifolia*) tree in 2010 from SERC follows.

doy.1 <- c(112, 120, 124,..., 301)dbh.1 <- c (18.99449, 18.99512, 19.00085,...,19.45890)

**Figure 2:**
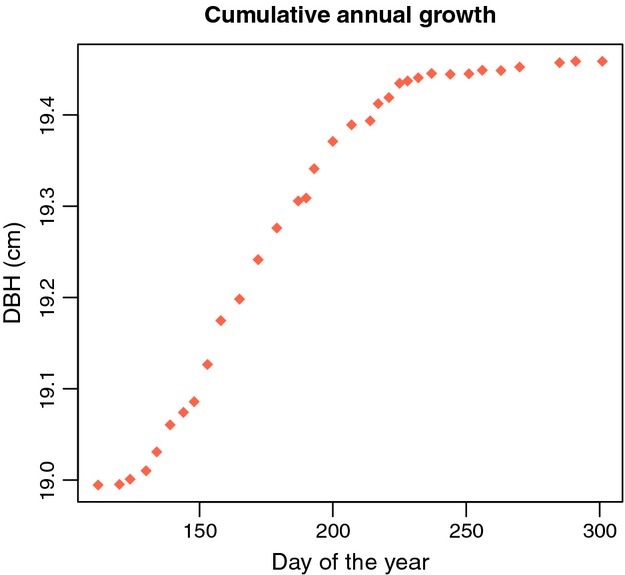
Typical data from an intensively measured dendrometer band. These data are of the first tree in the sample used in this paper. The data are of an extrapolated diameter (from the steel band and gap) on each day of the year (numbered from January 1) that measurements were taken.

Plotting these data shows the basic series of observations to which we will fit functional forms. Figure 2 demonstrates the detail that can be captured with weekly (or better) measurements.

### Models and comparisons

A family of functions, known collectively as Richards models, can produce good candidate parameterizations for intra-annual tree growth data (Oswald et al. 2012). Here we use a five-parameter logistic function. We found the effort to fit so many parameters worthwhile because the parameter number is important for precise fits, necessary to discover small fluctuations in growth during the year. The parameters have straightforward interpretations: two parameters define the lower and upper asymptotes (*L* and *K*, respectively), *doy*_*ip*_ marks the day of the year when the inflection of the curve is predicted to occur, and the rate parameter *r* describes the slope of the curve at the inflection point. The final parameter, *θ*, allows asymmetrical fits by changing the approach of the upper asymptote. This is a critical parameter as when *θ* = 1 the curve is symmetrical but growth forms are rarely symmetrical and so its inclusion merits the increased efforts in fitting a 5-parameter model. The model is then:

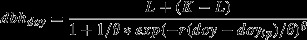
1

We created a predicting function from this equation (pred.lg5), which we use for optimization (see below). A major problem in fitting such high-parameter models is the flat- or complex-likelihood surface that cumulative growth data with five-parameter models can tend to provide. This is because there may be many combinations of parameters that can produce very similar fits. Our workflow solves this by proposing appropriate starting values and then honing the fit using a broad search before employing more focused search techniques.

### The optimization workflow

Figure 1 offers a graphical representation of the workflow. The data are collected (and transformed). Next we fit a model to the data, followed by several post hoc analyses that can return values from the data depending on the questions asked.

Fundamentally, we wanted a function to predict values of diameter given the day of the year 1. We use this predictive function to optimize the likelihood (minimizing the log-likelihood). For any parameter set *P*, the log-likelihood for data **x** is written as:

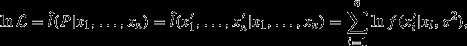
2

What this means in terms of the dendrometer band data is that for any proposed set of parameters (*P*) and the known days of the year which the actual data were taken (*doy*), (1) gives a predicted vector (time series) of dbh data (**x**^′^). The likelihood of these proposed dbh data comes from a normal probability density function with a mean vector *μ* = **x**, the observed dbh data, and a variance *σ*^2^ (which we set to 0.1^2^). More “likely” parameter sets (i.e., *L*, *K*, *doy*_*ip*_, *r*, and *θ*) produce predicted dbh data (**x**^′^ that is closer to the observed dbh data (**x**). The R function we use to return a negative log-likelihood value to the optimization algorithm follows:

>lg5.ML <- function(params, doy, dbh, resid.sd){pred.dbh <- lg5.pred(params, doy)pred.ML <- -sum(dnorm(dbh, pred.dbh, resid.sd,log = TRUE))return(pred.ML)}

Here the data are the day of the year (doy) and the observed dbh measurements (dbh), and the parameters (params) are proposed by the optimization function through its search algorithm. The lg5.pred function (which can be found in the code supplement) returns the predicted series of dbh measurements given proposed parameters and doy using (1). The pred.ML line takes the sum of the log-likelihood (as (2)) using the dnorm call, which is simply the probability density function a normal distribution (or in this case log-normal [note the “log = TRUE” argument]). resid.sd is the standard deviation used for the probability density function against which the data are tested (as stated above, we set this to *σ* = 0.1). When the optim() function is called, an optimization algorithm (we use a several algorithms in to develop our workflow) searches for optimal parameter values by minimizing the pred.ML value(see Bolker (2008) Chapter 7 for an excellent discussion of general maximum likelihood optimization using the R optim() function). An example of the optim() call looks like:

>lg5.output.NM <- optim(par = params, fn = lg5.ML,resid.sd = resid.sd,method = ''Nelder-Mead'', hessian = TRUE, doy = doy,dbh = dbh)

Here params are starting parameter values. The function to be minimized is lg5.ML, and other arguments for that function are given in the call. Other than the starting parameters, the “method” argument is the primary argument that we change. Under one algorithm, we add a “weighting” to the likelihood function that preferentially weights the extreme diameter measurements (see Appendix S1 for details).

In order to find an efficient protocol, we initially tested many combinations of potential algorithms (the methods argument for optim(), which Bolker (2008) Chapter 7 explains in detail), as well as different functional forms (not presented here, as we found the 5-parameter logistic function to be sufficiently flexible). Ultimately we found rules for starting values and boundaries (see Appendix S1) that supplied both reasonable starting values and then moved through all of the available optimization algorithms in optim() including using weighting functions for the early- and late-season diameter values. We then applied that protocol to growth data for 20 trees selected randomly from the data measured at SERC in 2010. Best models were chosen from the returned maximum likelihood values.

#### Starting values

Good starting values for parameter estimation are important primarily in order to avoid false maxima in the parameter surface (Bolker 2008). We proposed starting values for the data that reflect the complete lower and upper limits of diameters, and a positive slope. Negative slope (*r*) values can be produced by optim() when it also switches the lower asymptote value for the upper (the parameter *K* becomes the lower asymptote). This can create a fit equivalent to the positive *r*, but complicates comparing asymptote and slope values across. Our search protocol begins by exploring initial potential parameter values using the L-BFGS-B method (low-memory Broyden–Fletcher–Goldfarb–Shanno bounded algorithm), which allows lower and upper bounds on parameters and conducts a fairly broad search. We then used the parameters proposed by this method as starting parameters for the other functions. We did initially try to start every method with the base starting parameters, but these often produced very poor fits. When output from the bounded parameters were used instead, all of the algorithms performed consistently better. (Note that testing these assumptions simply requires changing some of the supplied R code and is an interesting exercise in optimization sensitivities.)

The methods we then applied to the Nelder-Mead algorithm, the BFGS algorithm (without lower and upper bounds), and a simulated annealing (SANN) search algorithm (using something similar to a Metropolis-Hastings algorithm that employs stochastic jump steps). We also ran all three of these algorithms using the maximum likelihood function with parameter weights to preferentially fit early and late diameter measurements.

### Diagnostics and post hoc procedures

#### Uncertainty

By running the optim() function with the argument hessian = TRUE, the output includes the hessian matrix. Using solve(model$hessian) returns the covariance matrix of the parameters. Adding and subtracting 2× the diagonal of this inverse hessian matrix to the parameter vector gives approximate 95% confidence intervals on the parameters.

#### Sample size analysis

We tested whether sample size might influence curve fits. To do this, we reran estimates of a single tree using fewer measurements of diameter spaced evenly in the interval between first and last measurements.

#### Upper and lower bounds

We also used the a separate likelihood test to search for potential minimum and maximum diameter sizes. We did this assuming that the fitted function was correct in estimating growth, but could not necessarily estimate accurate starting and ending diameter sizes. This makes our model a “semi-parametric” model or a threshold model (Bolker 2008). Our estimate of lower and upper diameter sizes connects directly to the optimal method selected in the fitting component. These values were retained as estimated diameters of trees at the beginning and end of the growing season, independent of the growth model parameters.

To estimate these values, we chose minimum and maximum values based on the data and the “best” model. Then, we ran a broad analysis of the best maximum likelihood fits of these lower and upper cutoff parameters. Because this exploratory analysis can be large, we refocused the search on parameter values within the expected range of the maximum (using a chi-squared test as with the profile log-likelihoods) and then estimated values within the new, focused constraints.

## Results

**Figure 3:**
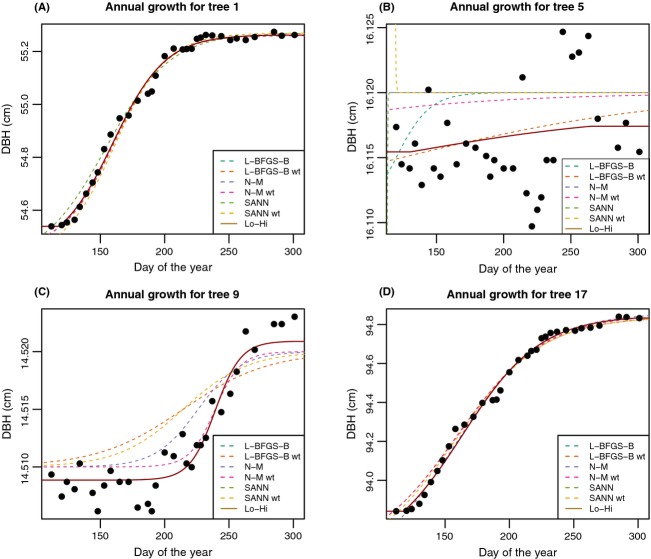
Four different trees fit with selected optimization arguments. Each tree was fit with the suite of optimization algorithms with the same starting parameter constraints. Solid red line shows the best fit (lowest AIC).

We found that all of the optim algorithms showed very similar fits, but only after starting parameter values were described by summary statistics and improved by a broad-bounded search (“L-BFGS-B”). After establishing this parameter set (following Fig. 1), the BFGS was rerun without bounds, along with fits for the data for four of the trees, including the beech tree (tree 1, Table1). Figures for all trees are included as Supplemental material. All fits were very similar for trees with high growth, and in fact all unweighted fits often tied with equivalent log-likelihoods (even as parameter values varied by small amounts). For 19 of the 20 trees, the Nelder-Mead method was either optimal or tied for optimal when another model had an identical likelihood. Only for tree nine (Fig. 3C), was there a different optimal method, which was BFGS weighted. Interestingly, this was the only methods that captured the high asymptote. We changed values for the starting parameters and bounding parameters, and reran the fits with exactly the same result, 19 of 20 optimized best or tied for best by Nelder-Mead, and tree nine optimized best by BFGS weighted.

### Uncertainty and sample size

Uncertainty was reasonably constrained for all parameter values for tree 1 (Fig. 5), and sample size had little effect on parameter estimates or uncertainty, except the inflection point (*doy*_*ip*_). This parameter had narrow uncertainty, but its value varied depending on sample size. Apparently small changes in data points number (and hence location) can lead to consistent values for all parameters except *doy*_*ip*_, which likely bounces around as the “free parameter.” The fit of this parameter encourages the use of other methods to estimate temporal patterns in these fits (see Day of starting and ending growth in the Discussion).

**Table 1:** Results from model runs using the 5-parameter logistic function. Parameter values are listed beside the optimization method. The asterisk in the final column refers to the best model

Optim.call	L	K	doy_ip	r	theta	ML	Best.ML
Starting	54.539	55.274	187	0.080	1.000	NA	NA
L-BFGS-B	54.402	55.270	187	0.044	3.655	−44.7814	0
L-BFGS-B wt	54.447	55.264	187	0.049	3.624	−44.7215	0
N-M	54.402	55.270	187	0.044	3.679	−44.7814	^*^
N-M wt	54.446	55.263	188	0.049	3.964	−44.7181	0
SANN	54.402	55.270	187	0.044	3.655	−44.7814	0
SANN wt	54.429	55.268	187	0.047	3.689	−44.7427	0

### Upper and lower bounds

We successfully fit consistent and satisfactory upper and lower limits of diameter (Table1). If the curve is long and smooth, these values will be similar or identical to the *L* and *K* parameters, but in many instances, for example, when growth begins abruptly, these will be different. Thus, it is important to fit the optimal growth process model first, and follow that fit with an estimate of upper and lower diameters.

## Discussion

We recommend using a series of optimization routines, each fed by new starting values. This effectively hones a search to achieve consistent results across a variety of growth forms. The first set of starting values was derived from summary statistics in the data, default values, and a series of lower and upper constraints (see code). These values were entered in a bounded L-BFGS-B search. The parameter estimates from this broad search were then used as starting values for the Nelder-Mead method. The only exception to this was when the weighted BFGS search captured important growth data in one tree (tree 9; Fig. 3C), which the Nelder-Mead method failed to capture. Unlike the SANN method, which is an MCMC search algorithm and can thus be computationally intensive, running the N-M and weighted BFGS methods is similarly fast. That said, a balance between finding the optimal model in each instance (in this case, one in twenty trees indicates a better fit from an algorithm other than the Nelder-Mead), and having easily comparable results must be weighed. This is discussed further below in the section Lower and upper bounds.

The maximum likelihood search for upper and lower bounds was fast, straightforward, and accurate. This fit fed easily into an algorithm to estimate high and low diameter values. The logistic optimization also established a solid beginning to a workflow that derived other important features of intra-annual tree growth.

### Residuals

**Figure 4:**
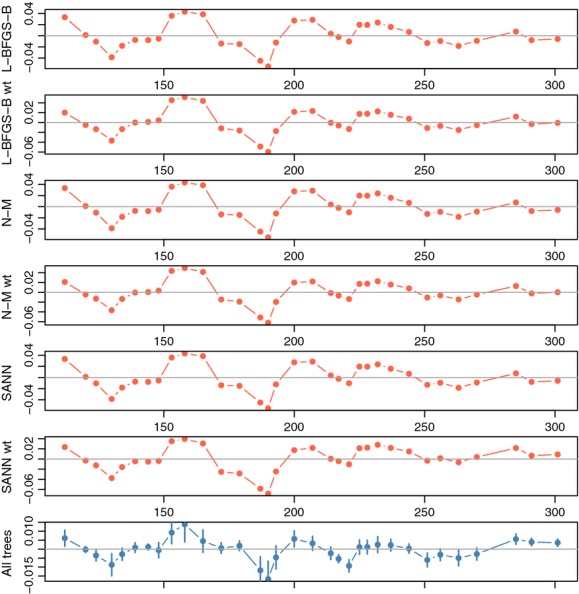
Residuals for the four LG5 models. The first six panels show residuals from the expected optimized model for a single tree (see Fig. 3A), and the last panel shows means of all 20 trees, with two standard errors from the mean shown in segments. Despite different species, locations, and sizes, the residuals show similar growth increment responses to weather during the growing season.

One of the goals of estimating a process model for these intra-annual growth data is to use the residuals of observed growth to infer the way in which a growth process might be affected by weather. The residuals for tree one differed very little across the different optimization algorithms (Fig. 4). The final fitted models captured the pattern of residuals across all 20 trees, as is evident in how the responses of trees around the fitted growth models are synchronized (Fig. 4 bottom panel). This synchronicity across trees indicates two important features of these fits: (1) that trees deviate similarly from their expected growth trajectories across the year (i.e., this is a good parametric function with which to capture growth), and (2) that the optimization of this function is consistent across data structures, tree size, and species. The synchronicity also indicates something of the phenomenon itself here. All of the trees are responding together–there is a signal of physiology in the growth response.

### Lower and upper bounds

Starting and ending diameter describes a tree's annual growth, the most common measurement derived from dendrometer bands. Although we are promoting an expansion of the use of dendrometer bands to model intra-annual growth, the total growth is an important value to have. In fact, our intra-annual measurements show that for many trees, annual or twice-annual measurements may not provide an accurate growth estimate. That is, the starting and ending diameter of a tree within a year is uncertain and is best estimated from a full season of data. We separated our estimation of the process (functional form) and the result (total growth) of diameter change, allowing the upper and lower asymptotes of the logistic function to vary independent of estimates of initial and final growth, freeing the logistic function to focus on the intra-annual growth process. Only after capturing the function of growth, did we search for starting and ending diameter values. For trees with substantial growth (such as tree 1 [Fig. 3A]), this may seem trivial, as the upper and lower diameter measurements align with a casual interpretation of total growth. However, trees with low growth (low signal to noise in the data) or abrupt starting and/or ending growth patterns will have asymptote parameters (*L* and *K*) that are outside the range of diameter data. Instead, we estimate two new parameters, *a* and *b*, So, we which describe the initial and final diameters in the year.

Estimating *a* and *b* uses the available diameter data as bounds for the low point and high point of the search. That is, we are not allowing initial or final diameters to lie outside of observed data. If multiple years of data have been collected, then estimates of *a* and *b* for adjacent years can also be used to inform these bounds, as information from other years may change this estimate. In estimating *a* and *b*, we are also accepting the fit of the optimized process model based on the lg5 function. So we do not rerun an optimization of the growth process model, but extend that model to have two potential threshold points, one at the beginning of the growing season and one at the end, that mark the shift from zero growth to seasonal growth and back to zero growth. Although this works well for most growth patterns, it may not always be appropriate.

If the functional form of the lg5 does not extend above high points or below low data points, these horizontal values will be excluded from optimizing the *a* and *b* parameters. For example, tree nine (Fig. 3C) shows that only the weighted BFGS model captures the upper asymptote well. Were we to rely on other models as the functional fit to these data, then our estimates for *b*, the maximum diameter at the end of the growing season, would fall significantly below the cloud of data that describe the late-season diameter measurements. This reminds us that using maximal fits from different methods may be important in some cases and that defaulting to only one method for efficiency can miss some special cases that have unique data structures. One can always take “problem” data and refit process model with new constraints, but for efficiency and consistency in estimating many trees, such deviations from optimal models can be simply accepted as fitting error. Such patterns, however, often occur for trees with low growth and therefore have small errors in estimates.

### Measurement frequency

**Figure 5:**
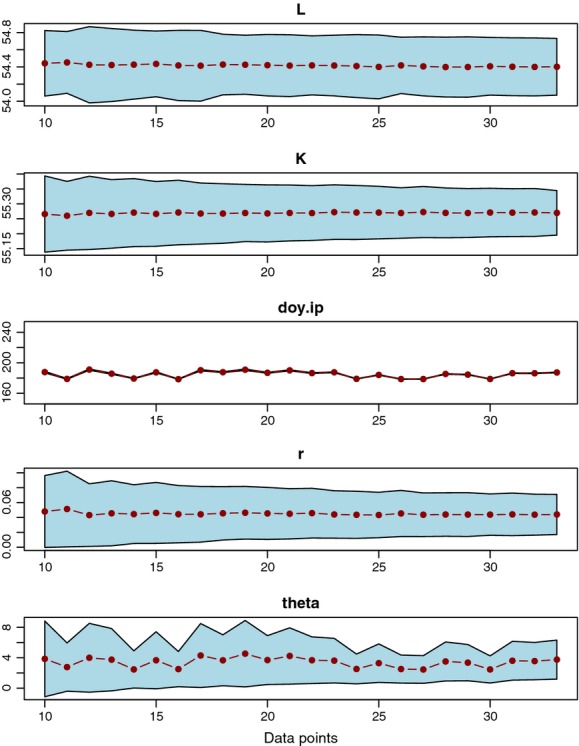
Parameters fit to different measurement frequencies. The five parameters from the output for tree number 1 are shown with 95% confidence intervals in blue. Connected dots show the parameter estimates. The x-axis shows the number of data used to fit the model, with 33 days of data being the full data set. In all sets, the first and the last days of data were retained and then remaining *N*−2 data points were retained in a roughly evenly distributed fashion (see Appendix S1 for details). Plots show that uncertainty is conserved across sample sizes, except for the inflection day (doy.ip), which has narrow CI, but changes for different fits.

The data used in this manuscript were collected from a protocol that required weekly measurements. This may not always be feasible and so we tested for changes in parameter estimates and parameter uncertainty when protocols sample regularly through the season, but with decreasing frequency. We found that for all parameters, fewer samples maintained good fits with high precision, except for *d*_*ip*_, which although precise in its estimation, changed around a range of values for each parameter fit (Fig. 5). This indicates that the inflection parameter acts somewhat as a “free parameter,” that moves within its range as the other parameters optimize to the different data patterns. This does not pose a serious concern because the fits all result in good models, and the *d*_*ip*_ is not used as an ecological metric (we instead use days of growth quantiles to investigate phenological changes in growth rates [see below]). Although measurement frequency does not influence model fit, it will influence inference about deviations from model fits and will influence optimization for noisier data sets or data from trees with little growth.

#### Implications of data from slow-growing trees

Annual growth increments that are smaller can approach both observational error and/or the deviations in growth due to weather or water volume in the trunk. Although growth estimates for these data are robust, function fits may lead to seasonal growth patterns that do not, visually, appear to reflect a reasonable growth model. If inference on the growth process for these trees is important to the research question, a more intensive measurement protocol may distinguish between observation error and growth changes.

One of the measured trees showed very little growth, giving us some insight into this observational error. The variance of the dbh data collected for tree five (Fig. 3B) is 0.03 mm. This estimate includes actual growth as well as some shrinkage in line with other trees in the forest. So, this estimate of error in measurements contains some process and growth variance as well, making it conservative, but illustrative of the precision of the process and the fitting approach. A simple repeated measurement of the same dendrometer band using different trees and repeated measurements by individuals would better identify this value in a particular study site.

In studies where comparison of the growth process across individuals, sites, and/or species is of interest, excluding or down-weighting these trees in analyses may be worth considering. For example, tree number five (Fig. 3B) shows that regardless of the accuracy of the fit, phenological values, such as the day of first, last, or 50% growth (see below for an explanation of how to derive these values), will add error to an analysis that compares these estimates to numbers derived from a clearer process model (such as for tree one [Fig. 3A]). We recommend excluding such outliers from synthetic analyses.

It is also worth noting that not all data are of a form amenable to this analysis. Data from less seasonal sites, such as the wet tropics, may need to be clipped to forms that best approximate annual growth. Also, trees with very slow growth will find that error in measurements approaches variance in measurements, and so no clear trend will appear. It is easy to see that with such slow growth, other methods to infer growth patterns, such as micro-cores, are preferred.

### Extensions for expanded inference

#### Day of starting and ending growth

Using the inverse of the logistic function (which is also used in the identification of upper and lower diameter bounds), it is easy to estimate the day of the year that certain growth values occur, such as the first day of growth and last day, or the day of fastest growth. The inverse function is:



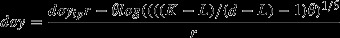
3

After fitting the function and obtaining values for the key parameters, one can use the inverse function to obtain a day of the year at which certain parameters have certain values. For example, using tree one (Fig. 3A), we estimate the start day as 118, the last day of growth as 289, and the day of 50% growth as day 164, or June 11th. Collected values of these dates can be used in analyses of phenology, physiology, or patterns of carbon allocation.

#### Maximum growth rate

The maximum growth rate in cm day^−1^ can be obtained using the derivative function lg5.deriv() folded into the function max.growth(). The max.growth() function uses the argument paras (which contain the five parameters of the logistic function given by the output from the optimization routines. For maximum growth rate, every day of the year is fed to the function (i.e., seq(365)). The maximum growth rate for tree one was 0.01, and this occurred on the day of the year 160.

#### Quantile-convex hull

One of the main features of modeling intra-annual growth is relating changes in growth rates to climate. Two primary factors determine intra-annual growth patterns: (1) the change in the allocation of carbon to bole growth (this might be termed “the fundamental growth rate”); and (2) the deviations from this functional form due to external influences (e.g., drought).

The process of intra-annual tree growth that we modeled using the logistic function allows us to estimate a progression of growth given diameter data. Factor (1) We have shown that using this fit and residual divergence from that fit can indicate how an individual tree, or the entire forest, responds to external influences. Factor (2) Although the residuals from this fit show a pattern of faster and slower growth due to weather, the residual values themselves are not the ideal way to quantify growth rates. This is, in this view, slower growth (negative residuals) shows a deviation from a maximum growth rate, but positive residuals do not equivalently reflect a faster than normal growth rate, but merely a return to normal growth. That is, slower growth is influenced by poor-growing conditions (e.g., high temperatures or drought), whereas high growth is due not to good conditions, but merely the lack of poor conditions. Therefore, we sought to extend our model of the growth process that uses information from all of the growth data (i.e., the optimal fit from the logistic function) but estimates the growth pattern that only allows deviations from normal growth. Essentially, we want to fit a type of convex hull to the data so that all residuals from that hull are negative.

**Figure 6:**
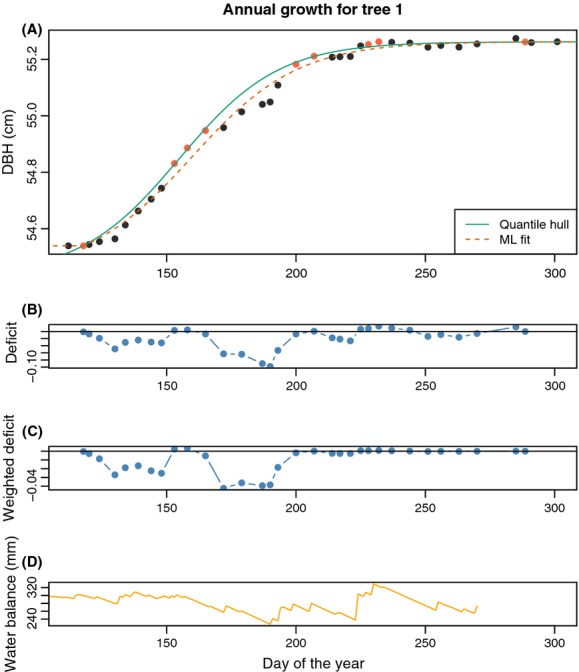
Quantile hull. This takes as endpoints the meeting of the ML fit with the asymptotes (high and low bounds) and then refits the LG5 to the points between these bounds that form the 80th quantile of the residuals (A). This allows us to keep the strengths of a parametric fit, as well as maintaining features of the fit of the total data. All residuals are now (mostly) negative, so that slower growth is isolated from the smooth process curve, allowing statistics on lags in growth. Lower panels show deviations from this growth trajectory both unweighted (B) and weighted by growth slope (C), and the annual water balance (D).

In fitting a model that captured the outer curve of growth, we did not want to lose the benefits of the logistic function (such as the derivative, rates, and parametric form), so we developed an approach we call the “quantile-convex hull” (QCH). This approach uses the parameters of the logistic function derived from all of the diameter data as a starting function, but is then refit using only the upper 80% quantile of the base model residuals. We cannot anticipate how a tree might have grown where it never perturbed, but the QCH offers insights into when and how severely growth deviated from the observed growth trajectory. Figure 6 shows the original fit, the QCH, and the data used to estimate it.

#### Weighted deviation from QCH

The QCH provides an estimate of the functional form of optimal intra-annual growth. Deviations from that growth form can be interpreted as the cessation of carbon allocation to growth or bole shrinkage. To tease apart mechanisms that might cause change in that allocation (such as high temperatures or drought) from the trunk water volume, one can use the deviations from the QCH in regression models. These deviations, or the sum of them over the year, should be weighted by the expectation of growth. To do this, we multiplied the deviations from the QCH by slope of expected growth at the time of measurement (i.e., the derivative of the QVH function). Panels b and c in Fig. 6 show the deviations from the hull in an unweighted and weighted, respectively. Low growth during times of the season where little growth is expected (such as late in the year) should not be as important to interpreting overall drought response as when growth is high. Summing the weighted deviations of each tree offers an index of annual deviation from growth. This value can be thought of as an integrated metric of growth over the year, facilitating comparisons between individual trees, species, plots, or years. The water balance graph (Fig. 6D) shows an estimate of available water over the growing season. The dips in the water balance line up with dips in the deviations from the outer hull, with earlier drops in available water showing a greater impact.

## Conclusions

We have detailed a pathway to use dendrometer band measurements of trees to capture an array of important features of tree growth. We hope that this effort will allow scientists currently measuring intra-annual growth to think about how these measurements can be used to infer processes such as carbon acquisition and allocation, and how deviations from growth models can be used to investigate features of forests important to large-scale vegetation models, such as drought response for different species. We also hope it will encourage ecologists who have dendrometer bands installed for annual censuses to measure them more often, as these intra-annual data can tell us a great deal about the physiological process of growth using an inexpensive tool (calipers) and a little bit of R code (Appendix S1).

## Supporting Information

**Appendix S1.** R code for all analyses.

**Appendix S2.** Forest_data.csv provides the data for analyses.

**Appendix S3.** WaterBalance.csv provides the data for analyses.
